# Comparison of Dentoalveolar Trauma Patterns Between E-Scooter and Bicycle Accidents in a German Urban Area: A Retrospective Study

**DOI:** 10.3390/dj13090409

**Published:** 2025-09-05

**Authors:** Anna Aydin, Johannes Schunk, Manfred Giese, Oliver Schuck, Florian Dudde

**Affiliations:** Department of Oral and Maxillofacial Surgery, Army Hospital Hamburg, Lesserstraße 180, 22049 Hamburg, Germany

**Keywords:** dentoalveolar trauma, patterns, e-scooter, bike

## Abstract

**Objectives:** The increasing popularity of electric scooters (E-scooters) has led to a rise in trauma-related injuries, particularly in the craniofacial region. This study aims to compare dentoalveolar trauma (DT) patterns between E-scooter and bicycle accidents in a German urban area to identify differences in injury characteristics, risk factors, and treatment approaches. **Materials and Methods:** This retrospective study analyzed 178 patients treated for DT following E-scooter (n = 56) or bicycle (n = 122) accidents between January 2022 and December 2022 at a single oral and maxillofacial surgery department in a German urban area. Demographic, clinical, temporal, and therapeutic data were collected and statistically compared. **Results:** E-scooter patients were significantly younger (mean age: 33.6 vs. 47.0 years, *p* < 0.001) and predominantly female (85.7% vs. 26.2%, *p* < 0.001) compared to bicycle patients. E-scooter accidents more frequently occurred at night (69.6%) and on weekends (62.5%) and were significantly associated with alcohol consumption (55.4%) and low helmet use (8.9%). Clinically, E-scooter accidents resulted in more complex dental injuries, including a higher incidence of root fractures (14.3% vs. 0%, *p* < 0.001), complicated crown fractures (25.0% vs. 9.0%, *p* = 0.004), and crown-root fractures (32.1% vs. 9.8%, *p* < 0.001). Mandibular injuries were more prevalent in the E-scooter group, and concomitant facial fractures (excluding alveolar) were also more common (28.6% vs. 19.7%, *p* = 0.018). **Conclusion:** E-scooter accidents are associated with a distinct DT pattern involving younger, often alcohol-intoxicated, and helmetless patients, typically presenting with more severe and complex DT-injuries. These findings highlight the urgent need for targeted prevention strategies, legislative measures on helmet use, and clinician awareness of specific trauma profiles linked to E-scooter use.

## 1. Introduction

Urban mobility has undergone a significant transformation in recent years, driven by increasing environmental awareness, traffic congestion, and the demand for flexible transport options [1]. Among the most notable innovations in this field is the emergence of electric scooters (E-scooters). Their ease of access, affordability, and integration into sharing systems have made them particularly attractive to younger populations and tourists [2]. However, this rapid adoption has been paralleled by a marked increase in accident rates and trauma-related emergency admissions [3,4].

Unlike bicycles, which benefit from decades of urban infrastructure planning, regulatory frameworks, and public safety campaigns, E-scooters remain largely unregulated in many regions [2,5]. This regulatory gap includes inconsistencies in helmet mandates, speed limitations, and designated traffic zones [2,5,6]. Additionally, the structural characteristics of E-scooters—such as small wheels, a high center of gravity, and front-loaded weight distribution—make them inherently unstable on uneven terrain. These mechanical vulnerabilities, combined with frequent nighttime use and the lack of protective equipment, contribute to a rising number of high-impact accidents, particularly involving the head and face [7,8]. Numerous studies have documented the increase in craniofacial trauma associated with E-scooter use [7,8,9]. However, specific analyses focusing on dentoalveolar trauma (DT)—which includes injuries to the teeth, periodontal structures, and alveolar bone—remain limited. DT is of particular clinical importance due to its immediate and long-term implications on oral function, aesthetics, and quality of life [10]. Untreated or improperly managed dental injuries can lead to tooth loss, chronic pain, infection, and psychosocial consequences [10,11]. Moreover, such injuries frequently require multidisciplinary management as well as long-term follow-up.

In contrast, the literature on bicycle-related DT is more established, reflecting the long-standing integration of bicycles into urban transportation networks in Central European countries such as Germany, Switzerland, Austria, and Italy [12,13]. While both E-scooters and bicycles share certain risk factors, such as exposure to vehicular traffic and vulnerability to falls, their user profiles, vehicle dynamics, and usage patterns may differ. E-scooter users, for example, often lack prior riding experience, are less likely to wear helmets, and may be more prone to intoxicated riding [14]. These factors may contribute to a distinct trauma mechanism and injury pattern compared to cyclists.

Given the lack of comparative studies, it remains unclear whether the frequency, and severity of DT differs significantly between E-scooter and bicycle accidents. A better understanding of these differences is essential not only for clinicians who manage acute trauma but also for public health officials.

To the best of our knowledge, no previous study has systematically compared dentoalveolar trauma patterns between E-scooter and bicycle accidents in a single cohort. While maxillofacial injury profiles of both vehicle types have been described separately, specific analyses focusing on dentoalveolar trauma remain scarce. This study therefore provides a novel contribution by identifying and contrasting distinct demographic profiles, risk factors, and clinical manifestations of dentoalveolar trauma between E-scooter and bicycle users. The findings are of particular necessity for both clinicians and public health authorities, as they inform tailored diagnostic and therapeutic approaches and support the development of targeted prevention strategies. By evaluating demographic variables, injury characteristics, temporal and seasonal patterns, and treatment modalities, this study aims to identify key differences that can inform clinical practice and enhance injury prevention.

## 2. Materials and Methods

This retrospective study included 178 patients who sustained DT following either an E-scooter or bicycle accident and were treated at the Department of Oral and Maxillofacial Surgery, Army Hospital Hamburg, between January 2022 and December 2022. Patients were allocated into two groups: those involved in E-scooter accidents (n = 56) and those involved in bicycle accidents (n = 122).

### 2.1. Data Collection

Data were extracted from patient records and included demographic information (age, gender), trauma-related parameters (type, number, and location of dental injuries), associated soft tissue and facial injuries, temporal distribution (day of the week and time of day), seasonal occurrence, alcohol consumption, helmet use, and treatment modalities. Exclusion criteria were patients with maxillofacial trauma without dentoalveolar involvement, incomplete records, and non-traumatic dental conditions. Patients presenting with jaw fractures (mandibular or midfacial) were excluded unless a concomitant dentoalveolar trauma was present. In such cases, the dentoalveolar injuries were analyzed as part of the study, and the associated jaw fractures were reported separately under “facial fractures”. These fractures (mandibular or midfacial) were classified according to standard maxillofacial trauma categories and treated following established guidelines, including open reduction and internal fixation or conservative management, depending on fracture type and displacement.

### 2.2. Classification of Trauma

Dentoalveolar trauma was classified according to the Andreasen system, which has been integrally adopted into the most recent WHO ICD-11 classification (NA0D—Injury of teeth or supporting structures). Accordingly, injuries were grouped into hard tissue and pulpal injuries (uncomplicated crown fractures, complicated crown fractures, root fractures), periodontal injuries (concussion, subluxation, extrusion, intrusion, lateral luxation, avulsion), and alveolar process fractures, the latter documented separately for analytical purposes. Treatment types included direct composite restorations, pulp capping, and splinting (semirigid or rigid).

### 2.3. Diagnostics, Treatment, and Postoperative Care

All patients underwent a standardized diagnostic work-up, including clinical examination by an oral and maxillofacial surgeon and radiographic imaging (panoramic radiograph and/or periapical radiographs; cone-beam computed tomography in selected complex cases). Treatment modalities were chosen according to the type and severity of dentoalveolar trauma. Uncomplicated crown fractures were primarily restored with direct composite restorations. Complicated crown fractures were managed with pulp capping or partial pulpotomy when indicated. Root and crown–root fractures were stabilized with splints (semirigid titanium trauma splints [TTS] or rigid splints in cases of extensive mobility). Luxation injuries were repositioned and subsequently splinted in line with established trauma guidelines. Postoperative management included antibiotic prophylaxis in cases of alveolar fractures or extensive soft tissue involvement, tetanus vaccination when indicated, and pain control as required. Patients with alveolar process fractures were followed up at our department until consolidation was achieved. For all other dentoalveolar injuries, postoperative care and long-term follow-up (including pulp vitality testing and definitive restorative or endodontic treatment) were delegated to the patients’ general dentists.

### 2.4. Statistical Analysis

Statistical analysis was conducted using SPSS Statistics (IBM, version 29.1, Markham, ON, Canada). Categorical variables were reported as absolute values and percentages, whereas continuous variables were expressed as mean ± standard deviation (SD). Group comparisons between E-Scooter and bicycle accidents were performed using Chi-square tests for categorical variables and independent t-tests for continuous variables. A *p*-value < 0.05 was considered statistically significant.

## 3. Results

A total of 178 patients were included in the analysis: 56 (31.5%) patients sustained trauma via E-scooter accidents, and 122 (68.5%) patients via bicycle accidents (Table 1). The mean age of E-scooter users was significantly lower (33.64 ± 10.74 years) than that of cyclists (46.97 ± 11.60 years, *p* < 0.001) (Table 1). Female patients were significantly overrepresented in the E-scooter group (85.7%) compared to the bicycle group (26.2%, *p* < 0.001) (Table 1).

### 3.1. Dentoalveolar Trauma Characteristics

**Figure 1:**
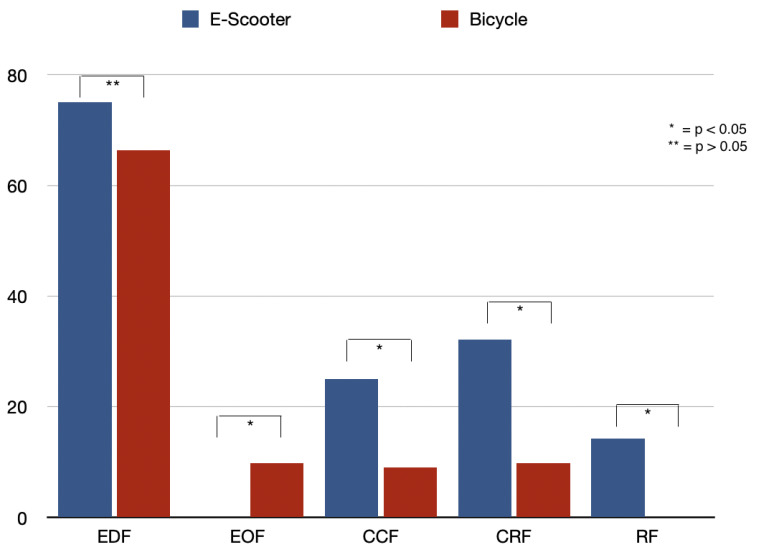
Fracture type relative distribution. Note: EDF = Enamel + dentin fracture, EOF = Enamel only fracture, CCF = Complicated crown fracture, CRF = Crown root fracture, RF = Root fracture.

The average number of affected teeth was significantly lower in the E-scooter group (1.79 ± 0.95) than in the bicycle group (2.30 ± 1.70, *p* = 0.034) (Table 2). Complicated crown fractures (25.0% vs. 9.0%, *p* = 0.004), crown-root fractures (32.1% vs. 9.8%, *p* < 0.001), and root fractures (14.3% vs. 0%, *p* < 0.001) were more prevalent among E-scooter patients (Table 2, Figure 1). Lateral luxation occurred exclusively in the bicycle group (18.0%, *p* < 0.001), whereas subluxation and extrusion showed no significant group differences (Table 2). Combined tooth fractures and dislocations were more common in E-scooter patients (80.4% vs. 63.9%, *p* = 0.028) (Table 2). Alveolar fractures occurred in 7.1% of E-scooter patients and 9.8% of cyclists (*p* = 0.560) (Table 2). The most frequently affected region in both groups was the maxillary incisor area (E-scooter: 85.7%, bicycle: 80.3%, *p* = 0.385) (Table 2). Mandibular injuries were more common in the E-scooter group, particularly to the mandibular left molar (14.3% vs. 0%, *p* < 0.001) and premolar regions (7.1% vs. 0%, *p* = 0.003) (Table 2).

### 3.2. Alcohol Consumption and Helmet Use

Alcohol consumption was significantly more common among E-scooter users (55.4%) than cyclists (34.4%, *p* < 0.001) (Table 2). Helmet usage was significantly lower in the E-scooter group (8.9%) compared to the bicycle group (48.4%, *p* < 0.001) (Table 2).

### 3.3. Temporal and Seasonal Patterns

**Figure 2:**
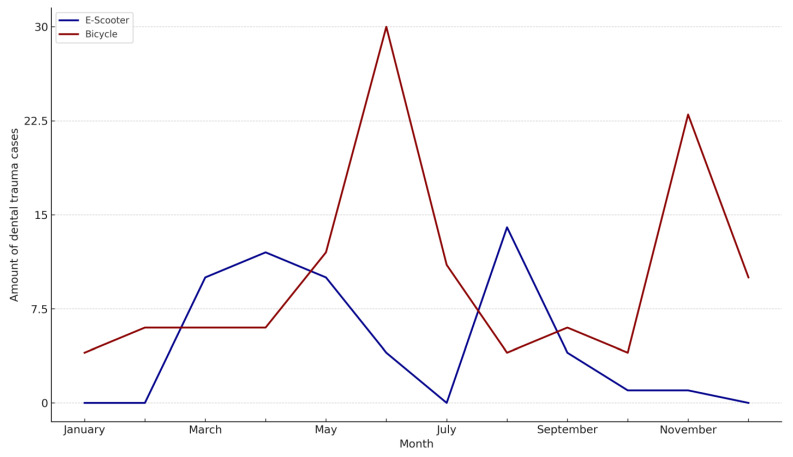
Distribution of monthly accidents.

E-scooter-related injuries occurred more frequently on weekends (62.5%) and during nighttime hours (69.6%), whereas bicycle injuries were more evenly distributed over the week and predominantly occurred in the morning and evening hours (*p* < 0.001) (Table 3). E-scooter accidents peaked in March, April, May, and August, while bicycle-related accidents were more evenly distributed, with a notable peak in June and November (Table 3, Figure 2).

### 3.4. Treatment

Composite restorations were more frequently used in the bicycle group (85.2%) than in the E-scooter group (69.6%, *p* = 0.015) (Table 4). Semirigid splints were applied more often in E-scooter patients (69.6% vs. 59.0%, *p* = 0.017), while rigid splint usage was comparable between groups (*p* = 0.383) (Table 4). Pulp capping was significantly more frequent in E-scooter patients (28.6% vs. 18.9%, *p* = 0.014) (Table 4).

## 4. Discussion

The present retrospective study aimed to analyze and compare the patterns of DT sustained in (E-scooter versus bicycle accidents. The findings highlight distinct demographic, temporal, clinical, and therapeutic differences between both groups, thereby underlining the need for differentiated preventive and diagnostic approaches.

One of the most striking differences observed was the significantly younger average age in the E-scooter group compared to the bicycle group. E-scooter riders were, on average, more than a decade younger, which is consistent with current usage trends, where younger adults and adolescents are the predominant user group for E-scooters [5,15]. Furthermore, a clear female predominance was observed among E-scooter patients, contrasting sharply with the male predominance in bicycle trauma cases. This suggests sex-specific usage patterns that may influence trauma risk profiles and should be considered in public health campaigns and protective gear marketing.

E-scooter accidents were more frequent during weekends and nighttime hours, while bicycle-related trauma was more evenly distributed across the week and more likely to occur during morning and evening hours. These differences could reflect the recreational and often spontaneous use of E-scooters, which tend to be utilized more during leisure times and under potentially impairing conditions such as poor lighting or intoxication [14,16]. This hypothesis is supported by the significantly higher incidence of alcohol consumption among E-scooter users. In contrast, bicycle usage is often part of daily routines (e.g., commuting), potentially explaining the observed distribution [17]. Interestingly, E-scooter-related trauma peaked in spring and late summer months, while bicycle accidents showed a more stable distribution with peaks in early summer and late autumn. This seasonal discrepancy might be attributed to the novelty and trend-based nature of E-scooter usage, which may follow patterns of public events, tourism, and weather-related accessibility [18,19].

### 4.1. Trauma Patterns

Clinically, E-scooter-related dentoalveolar trauma exhibited a higher prevalence of complicated injuries, including crown-root fractures, complicated crown fractures, and root fractures. These injury types suggest a more forceful impact mechanism, possibly due to the inherent instability of E-scooters, especially at higher speeds or on uneven surfaces [16,20]. In addition, the lack of protective infrastructure (e.g., bike lanes) and sudden deceleration from front-heavy designs may predispose riders to facial impacts [4,21]. Contrarily, bicycle trauma was more frequently associated with lateral luxation and enamel-only fractures, indicating a different kinetic profile with potentially more glancing impacts. Notably, combined injuries (tooth fracture + dislocation) were more prevalent in the E-scooter group, reinforcing the hypothesis of more severe traumatic forces.

Mandibular trauma, especially to the left molar and premolar regions, was significantly more common among E-scooter riders. This could be due to fall dynamics, where the rider’s lower jaw is more exposed during direct facial impact. Additionally, the near absence of mandibular involvement in the bicycle group underscores the differences in fall dynamics. Another noteworthy finding was the predominance of left-sided tooth injuries. This asymmetry may be explained by instinctive protective mechanisms during falls. Biomechanical studies suggest that individuals often deviate or turn toward their dominant or “working” side to protect vital structures, which may unintentionally expose one side of the dentition to higher impact forces. Such reflexive movements could help explain the observed left-sided predominance in our cohort.

### 4.2. Associated Injuries and Risk Factors

The rates of concomitant soft tissue lacerations were comparable between both groups; however, facial fractures (excluding alveolar fractures) were significantly more prevalent in E-scooter users. This finding underscores the higher energy transfer in E-scooter accidents and the possible involvement of additional craniofacial structures [22,23].

Helmet usage was dramatically lower in the E-scooter group, which likely contributed to the increased incidence of complex trauma. Unlike bicycles, where helmet promotion has a longer tradition and legal enforcement in some jurisdictions, E-scooters often lack clear safety regulations and user education [15,24]. Public health policies must address this disparity, potentially through mandatory helmet laws or integrated safety features.

The high prevalence of alcohol use among E-scooter patients (over 55%) presents a critical and modifiable risk factor. While cycling under the influence is also dangerous, the spontaneity and low barrier to access E-scooters make intoxicated riding more likely. Legislative measures, technological safeguards (e.g., breathalyzer locks), and awareness campaigns may solve this deficit. Beyond the dentoalveolar and craniomaxillofacial region, E-scooter and bicycle accidents frequently result in concomitant injuries to other organ systems. Previous reports have described a considerable rate of associated concussions in patients sustaining maxillofacial trauma, underlining the importance of a structured neurological assessment in the acute setting [25]. In addition, extremity fractures and thoracic contusions are among the most common systemic injuries reported in larger trauma cohorts of micro-mobility accidents [4,26]. While these injuries were not the primary focus of the present study, they illustrate the high-energy mechanisms involved and the need for interdisciplinary management. From a surgical perspective, soft tissue lacerations represent the most frequent maxillofacial concomitant injury and carry the risk of secondary infection, which must be addressed by appropriate wound management and, if indicated, antibiotic prophylaxis. Standard trauma protocols also include tetanus prophylaxis in cases with contaminated wounds. These considerations emphasize that dentoalveolar trauma rarely occurs in isolation and should always be evaluated in the broader context of polytrauma care.

### 4.3. Treatment Modalities

From a therapeutic standpoint, E-scooter patients were more likely to receive semirigid splints and pulp capping, while composite restorations were more common among bicycle patients. These findings are consistent with the observed DT injury severity, where more complex injuries required stabilizing and biologically conservative measures. The type of trauma guides clinical decision-making, and the differences in treatment reflect appropriate standards of care. The need for interdisciplinary treatment, involving oral and maxillofacial surgeons, restorative dentists, and endodontists, is evident, especially in high-energy trauma cases [27]. Establishing structured trauma pathways and documentation protocols could enhance long-term outcomes.

### 4.4. Current Knowledge and Research Gap

Several recent studies have described the increasing incidence of maxillofacial injuries related to E-scooter use, often reporting high rates of craniofacial trauma, alcohol intoxication, and low helmet use. However, these investigations predominantly focus on overall facial fractures or emergency department admissions, while detailed analyses of DT remain scarce. In contrast, bicycle-related DT has been more extensively documented over the past decades, reflecting the long-standing role of cycling in urban mobility. Nevertheless, most bicycle studies examine trauma in isolation, without direct comparison to emerging transport modalities such as E-scooters. As a result, little is known about whether E-scooter accidents lead to a distinct DT profile compared to bicycle accidents. The present study is, to the best of our knowledge, the first to directly compare DT sustained from E-scooter and bicycle accidents in a single cohort. By systematically analyzing demographic variables, injury patterns, temporal factors, and treatment approaches, we were able to demonstrate clear differences between the two groups. Specifically, E-scooter accidents were associated with younger, predominantly female patients, higher rates of alcohol consumption, lower helmet use, and more complex injury types, including crown-root and root fractures as well as mandibular involvement. These findings underscore that E-scooter-related DT represents a unique clinical entity with distinct epidemiological and therapeutic implications.

### 4.5. Limitations and Future Perspectives

This study is limited by its retrospective design and potential underreporting or misclassification bias. The sample originates from a single trauma center, which may limit generalizability. Furthermore, not all confounders (e.g., helmet type, fall height, rider experience, alcohol level) were controlled. Another limitation is that the analysis was restricted to the year 2022. This choice was deliberate to ensure a complete 12-month cycle with seasonal variation based on fully validated records. At the time of data extraction and statistical analysis, subsequent years (2023–2024) were either incomplete or still undergoing verification in the hospital’s documentation system. Future studies should therefore extend the observation period to multiple years to confirm the stability and reproducibility of our findings. Future prospective studies should aim to validate these findings across multiple centers and include radiographic, functional, and quality-of-life outcomes. Additionally, biomechanical analyses and simulations may shed light on specific impact mechanisms, thereby supporting the development of targeted protective measures.

## 5. Conclusions

This study demonstrates that DT sustained from E-scooter accidents presents with distinct epidemiological and clinical characteristics compared to bicycle-related DT. E-scooter incidents were associated with younger, predominantly female patients, higher rates of alcohol consumption, lower helmet usage, and a greater incidence of complex dental and facial injuries. These findings suggest a more severe trauma mechanism in E-scooter accidents, often occurring during nighttime and recreational activities.

Importantly, the predominance of young, often intoxicated female riders during nighttime accidents highlights that demographic and behavioral factors alone cannot fully explain the observed differences. Comparable populations using conventional bicycles did not show the same extent of dentoalveolar trauma. This suggests that intrinsic risk factors of E-scooters—such as higher speed, lower vehicle stability, and limited facial protection during impact—play a decisive role. In addition, sex-related differences in physical strength and protective reflexes may contribute to the increased vulnerability of female riders in high-speed impacts, resulting in more severe DT outcomes.

Given the increasing popularity of E-scooters and the severity of associated injuries, targeted public health strategies are urgently needed. These might include mandatory helmet legislation, educational campaigns on safe E-scooter usage, and regulations addressing intoxicated riding. Clinicians should be aware of the characteristic injury patterns to optimize diagnostic and therapeutic approaches. Future research should aim at prospective, multicenter studies with larger cohorts to validate our findings. In addition, biomechanical analyses and long-term follow-up investigations are warranted to better understand trauma mechanisms, treatment outcomes, and to inform preventive strategies.

## Figures and Tables

**Table 1:** Patient’s Characteristics.

Variable	Total (n = 178)	E-Scooter (n = 56)	Bicycle (n = 122)	*p*-Value
Age (years)	42.78 (±12.90)	33.64 (±10.74)	46.97 (±11.60)	**<0.001**
Gender				**<0.001**
Male	98 (55.1)	8 (14.3)	90 (73.8)	
Female	80 (44.9)	48 (85.7)	32 (26.2)	

Note: Data presented as mean (SD) and/or absolute values (percentage).

**Table 2:** Dentoalveolar trauma characteristics.

Variable	Total (n = 178)	E-Scooter (n = 56)	Bicycle (n = 122)	*p*-Value
Amount of Teeth	2.14 (± 1.52)	1.79 (± 0.95)	2.30 (± 1.70)	**0.034**
Enamel + Dentin Fracture (Uncomplicated Crown Fracture)	123 (69.1)	42 (75.0)	81 (66.4)	0.249
Crown Root Fracture	30 (16.9)	18 (32.1)	12 (9.8)	**<0.001**
Complicated Crown Fracture	25 (14.0)	14 (25.0)	11 (9.0)	**0.004**
Enamel Fracture Only (Uncomplicated Crown Fracture)	12 (6.7)	0 (0)	12 (9.8)	**0.015**
Root Fracture	8 (4.5)	8 (14.3)	0 (0)	**<0.001**
Concussion	47 (26.4)	13 (23.2)	34 (27.9)	0.513
Subluxation	47 (26.4)	20 (35.7)	27 (22.1)	0.056
Palatinal Luxation	54 (30.3)	16 (28.6)	38 (31.1)	0.728
Avulsion	43 (24.2)	14 (25.0)	29 (23.8)	0.859
Extrusive Luxation	11 (6.2)	6 (10.7)	5 (4.1)	0.089
Lateral Luxation	22 (12.4)	0 (0)	22 (18.0)	**<0.001**
Intrusive Luxation	23 (12.9)	6 (10.7)	17 (13.9)	0.552
Combined Tooth Fracture + Dislocation	123 (69.1)	45 (80.4)	78 (63.9)	**0.028**
Alveolar Fracture	16 (9.0)	4 (7.1)	12 (9.8)	0.560
Maxillary Left Molar	6 (3.4)	0 (0)	6 (4.9)	0.091
Maxillary Left Premolar	0 (0)	0 (0)	0 (0)	**/**
Maxillary Left Caninus	10 (5.6)	4 (7.1)	6 (4.9)	0.549
Maxillary Incisor	146 (82.0)	48 (85.7)	98 (80.3)	0.385
Maxillary Right Caninus	12 (6.7)	0 (0)	12 (9.8)	**0.015**
Maxillary Right Premolar	0 (0)	0 (0)	0 (0)	**/**
Maxillary Right Molar	6 (3.4)	0 (0)	6 (4.9)	0.091
Mandibular Left Molar	8 (4.5)	8 (14.3)	0 (0)	**<0.001**
Mandibular Left Premolar	4 (2.2)	4 (7.1)	0 (0)	**0.003**
Mandibular Left Caninus	6 (3.4)	0 (0)	6 (4.9)	0.091
Mandibular Incisor	6 (3.4)	0 (0)	6 (4.9)	0.091
Mandibular Right Caninus	0 (0)	0 (0)	0 (0)	/
Mandibular Right Premolar	0 (0)	0 (0)	0 (0)	/
Mandibular Right Molar	10 (5.6)	4 (7.1)	6 (4.9)	0.549
Combined Mandibular + Maxillary Teeth	6 (3.4)	0 (0)	6 (4.9)	0.091
Concomitant Soft Tissue Laceration	132 (74.2)	42 (75.0)	90 (73.8)	0.862
Concomitant Facial Fracture (excl. Alveolar Fracture)	40 (22.5)	16 (28.6)	24 (19.7)	**0.018**
Alcohol Consumption	73 (41.0)	31 (55.4)	42 (34.4)	**<0.001**
Helmet Use	64 (35.9)	5 (8.9)	59 (48.4)	**<0.001**

Note: Data presented as mean (SD) and/or absolute values (percentage).

**Table 3:** Distribution of dentoalveolar trauma.

Variable	Total (n = 178)	E-Scooter (n = 56)	Bicycle (n = 122)	*p*-Value
Weekend	89 (50.0)	35 (62.5)	54 (44.3)	**0.024**
Weekday	89 (50.0)	21 (37.5)	68 (55.7)	**0.024**
Morning	44 (24.7)	4 (7.1)	40 (32.8)	**<0.001**
Evening	74 (41.6)	13 (23.2)	61 (50.0)	**<0.001**
Night	60 (33.7)	39 (69.6)	21 (17.2)	**<0.001**
Month				**<0.001**
January	4 (2.2)	0 (0)	4 (3.3)	
February	6 (3.4)	0 (0)	6 (4.9)	
March	16 (9.0)	10 (17.9)	6 (4.9)	
April	18 (10.1)	12 (21.4)	6 (4.9)	
May	22 (12.4)	10 (17.9)	12 (9.8)	
June	34 (19.1)	4 (7.1)	30 (24.6)	
July	11 (6.2)	0 (0)	11 (9.0)	
August	28 (15.7)	14 (25.0)	4 (3.3)	
September	10 (5.6)	4 (7.1)	6 (4.9)	
October	5 (2.8)	1 (1.8)	4 (3.3)	
November	24 (13.5)	1 (1.8)	23 (18.9)	
December	10 (5.6)	0 (0)	10 (8.2)	

Note: Data presented as absolute values (percentage). Morning = 8 a.m.–4 p.m.; Evening = 4 p.m.–12 p.m., Night = 12 a.m.–8 a.m.

**Table 4:** Treatment of dentoalveolar trauma.

Variable	Total (n = 178)	E-Scooter (n = 56)	Bicycle (n = 122)	*p*-Value
Composite	143 (80.3)	39 (69.6)	104 (85.2)	**0.015**
Pulp capping	39 (21.9)	16 (28.6)	23 (18.9)	**0.014**
Semirigid Splint (TTS)	111 (62.4)	39 (69.6)	72 (59.0)	**0.017**
Rigid Splint	20 (11.2)	8 (14.3)	12 (9.8)	0.383

## Data Availability

The datasets generated and/or analyzed during the current study are not publicly available due to privacy and data protection regulations but are available from the corresponding author on reasonable request.
